# Unraveling the mystery of hearing in gerbil and other rodents with an arch-beam model of the basilar membrane

**DOI:** 10.1038/s41598-017-00114-x

**Published:** 2017-03-22

**Authors:** Santosh Kapuria, Charles R. Steele, Sunil Puria

**Affiliations:** 1 Department of Applied Mechanics, Indian Institute of Technology Delhi, New Delhi, 110016 India; 2 Stanford University Department of Mechanical Engineering, Stanford, CA 94305 USA; 3 Stanford University Department of Otolaryngology–Head and Neck Surgery, Stanford, CA 94305 USA

## Abstract

The mammalian basilar membrane (BM) consists of two collagen-fiber layers responsible for the frequency-to-place tonotopic mapping in the cochlea, which together form a flat beam over at least part of the BM width. The mechanics of hearing in rodents such as gerbil pose a challenge to our understanding of the cochlea, however, because for gerbil the two layers separate to form a pronounced arch over the remaining BM width. Moreover, the thickness and total width normally thought to determine the local stiffness, and tonotopic mapping in turn, change little along the cochlear length. A nonlinear analysis of a newly developed model, incorporating flat upper and arched lower fiber layers connected by ground substance, explains the initial plateau and subsequent quadratic increase found in measured stiffness vs. deflection curves under point loading, while for pressure loading the model accurately predicts the tonotopic mapping. The model also has applicability to understanding cochlear development and to interpreting evolutionary changes in mammalian hearing.

## Introduction

As a prism separates white light into a spectrum of colors, the cochlea of the mammalian inner ear acts to mechanically separate an incoming sound signal into component frequencies. It is well established that this frequency analysis results from a process called tonotopic mapping^1^, in which each frequency component induces a maximum mechanical vibration at a different place along the cochlear length. As a result, higher frequencies excite sensory cells at the basal end of the cochlea near the stapes, while lower frequencies excite sensory cells toward the apical end near the helicotrema. Almost every structural component of the cochlea has, at one time or another, been proposed as a key resonance element responsible for this frequency-to-place cochlear mapping. However, the dominant view, backed by direct measurements pioneered by Nobel laureate Georg von Békésy^2^, is that the basilar membrane (BM), the structure that varies the most dramatically along the length of the cochlea, is the best candidate.

**Figure 1:**
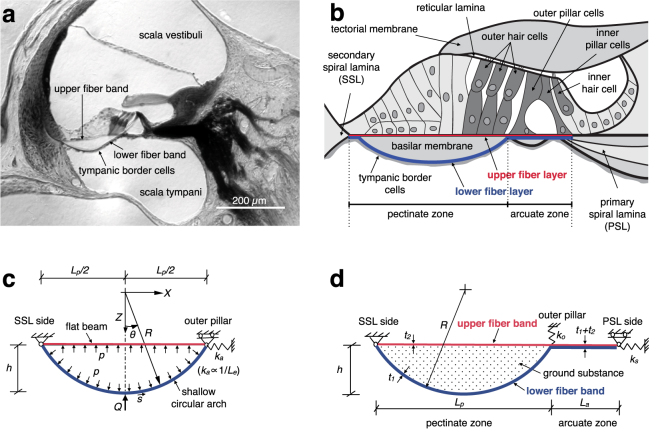
(**a**) Image of a gerbil cochlea, showing the large arch-shaped lower fiber band and flat upper fiber band of the BM^11^. Also visible are the tympanic border cells (TBCs) attached to the underside of the arch. (**b**) A schematic cross section of the gerbil BM and organ of Corti, showing the pectinate zone (PZ) and arcuate zone (AZ). (**c**) A simplified version of the arch–beam model (ABM) used to determine parameters of the arch without the AZ beam. Model parameters shown are point-load Q, internal pressure p, and spring stiffness *k*_*a*_, representing the in-plane stiffness of the AZ and the PSL. The radius of curvature *R* determines the degree of separation between the two fiber layers in the PZ. (**d**) Geometry of the full ABM. Model parameter *k*_*s*_ represents the in-plane stiffness for radial displacement of the PSL and SSL and *k*_*o*_ represents the outer-pillar support stiffness.

The BM is an acellular membrane composed of stiff radially oriented collagen fibers, embedded in soft ground substance, that span the varying width along the length of the cochlea between the primary spiral lamina (PSL) and secondary spiral lamina (SSL; Fig. 1a,b). These fibers form a single layer between the PSL and outer pillar cell, known as the arcuate zone (AZ); whereas from the outer pillar cell to the SSL, known as the pectinate zone (PZ), the same fibers separate to form an upper and lower layer with intervening ground substance, as noted from investigations of the BM ultrastructure in guinea pig^3^, cat^4^, and gerbil^5^. On its lower surface facing the scala tympani (Fig. 1a,b), the BM is covered by a layer of soft tympanic border cells (TBCs)^5–8^.

An unusual characteristic of the PZ in the gerbil (as well as in the mouse and moles of the genus *Talpa* ^9^), is that it contains much more ground substance between the upper and lower fiber layers than in other mammals^10–13^, such that the PZ forms a distinctively arched shape (Fig. 1a,b) in these species^5, 9, 13, 14^.

Variation in the local volume stiffness (pressure/volume-displacement) of the BM along its length is the primary reason for the frequency–place mapping of the BM, given that the local resonance frequency is proportional to the square root of this local stiffness. Therefore, one can estimate the range of stiffness of the BM in a given species by considering the range of frequencies that the species is capable of hearing (see Table S1).

Mathematical models used for quantifying the BM response typically consider the upper and lower collagen-fiber layers of the BM as a single layer with homogeneous material properties as in the simple beam model (SBM)^15–20^. The resonance frequencies computed using the SBM (see Methods Eq. 1), in conjunction with measured anatomical dimensions, have been shown to predict the frequency range of hearing reasonably well for several mammals^21^. In the guinea pig (GP), for example, the SBM predicts a base/apex resonance-frequency ratio of 400 (Table S1). This is 43% of the actual ratio of 926. When the same is attempted for the Mongolian gerbil, however, the SBM predicts a base/apex ratio of only 2.7 in the resonance frequencies, which is a mere 0.45% of the actual ratio of 600.

The discrepancy in SBM predictions between the GP and gerbil is related to how the gerbil’s BM thickness is not uniform across its width, due to the prominent PZ arch, and how this affects measurements of its thickness. Whereas the relatively flat BM of the GP decreases in thickness by nearly a factor of 7 from base to apex, i.e., from around 7 μm to 1 μm, in the gerbil the BM thickness measurements with an arched PZ actually increase from 22.6 μm at the base to 56.5 μm at the apex, corresponding to a base/apex ratio of only 0.4 (Table S1). The base/apex PZ-width change is comparable in both species, the base/apex local fiber volume fraction ratio in the GP is about 0.4 times the value in the gerbil, and the fluid density is assigned the same value for both species. Given this, the SBM predicts that the base/apex resonance-frequency ratio for GP should be around 45–148 times higher than for gerbil. This brings into question how the thickness of the BM should be accounted for in mathematical models when the PZ features an arch^5, 13, 14, 22, 23^.

There have been many attempts to measure the stiffness of the gerbil BM under a direct mechanical point load (force/displacement), such as those made by research groups at Boston University^6, 22^ and Northwestern University^24^. For one, a 41-fold change in the base/apex BM point-load stiffness of the Mongolian gerbil was reported by Naidu and Mountain of the Boston group^6, 22^. This is far below the expected point-load stiffness ratio, which should be around four orders of magnitude (~5000) to support the normal gerbil hearing range. Emadi *et al.* of the Northwestern group came somewhat closer to the expectation with a measured stiffness change of 335-fold^24^.

However, these point-load stiffness measurements as a function of increasing deflection made by both groups exhibit some unusual features, namely: (i) a long initial plateau of low stiffness, (ii) a sudden rise in stiffness, (iii) a local minimum often referred to as the ‘second plateau’ in the literature, and then (iv) a quadratic increase in stiffness for the highest point deflections. It has so far been argued that the second plateau in these stiffness–deflection curves represents the stiffness of the BM that determines the frequency–place map of the gerbil when measured at low levels^6, 17, 22^, even though the second plateau occurs at a deflection in the 10–20 μm range, which is much higher than the normal <1 μm physiological motion of the BM of a healthy cochlea^24^. Under point loads causing a deflection of more than a few thicknesses of the lower arch layer, the arch will begin to buckle. Therefore, the reported second-plateau stiffness measurements are well into the nonlinear buckling region. How then is it meaningful to use the second plateau as the physiologically relevant stiffness?

To elucidate the gerbil point-load stiffness measurements and to provide insights into the cochlear map for the gerbil, a new model has been developed that takes into account the actual geometry of the two collagen-fiber layers, treating the upper layer as a flat beam and the lower layer as an arch in the PZ, as shown in Fig. 1c. The beam and arch are coupled by ground substance, modeled as an incompressible gel. In the AZ (Fig. 1d), the two fiber layers merge to form a beam of combined thickness. This formulation is termed the ‘arch–beam model’ (ABM).

One feature of the point-load stiffness measurements is that the loading and unloading data show different stiffness curves at small probe deflections^8, 22^. The unloading curve reveals a characteristic sign of buckling behavior of the lower arch that cannot be explained by the SBM. The ABM for the point-load stiffness measurements accounts for the large strain–displacement geometric nonlinearity and buckling, and includes the effects of the layer of soft TBCs. It is presently shown that incorporating this TBC cover layer is critical for re-interpreting the previously measured loading and unloading measurements.

The ABM has also been adapted to the case in which the gerbil BM is loaded via pressure in the surrounding fluid rather than a mechanical point load, to obtain the frequency–place cochlear mapping based on the linear volumetric stiffness, which is the relevant quantity for estimating the frequency–place cochlear map. The ABM is then applied to the mouse anatomy, which features a similar PZ arch structure, but with less curvature than in the gerbil. Another generalization of the ABM model is its possible extension to the gerbillinae subfamily and to the development of the BM after birth.

## Results

### Sensitivity of point-load and point-stiffness curves to the ABM parameters

Model responses were first obtained using a rigid outer-pillar support and without considering the effect of the TBCs (Fig. 1c), to test the sensitivity of the responses to the model parameters such as the volume dispersal factor *c*, the effective spring length at the arcuate zone end *L*_*e*_, and the elastic modulus *E* of the collagen-fiber bands.

**Figure 2:**
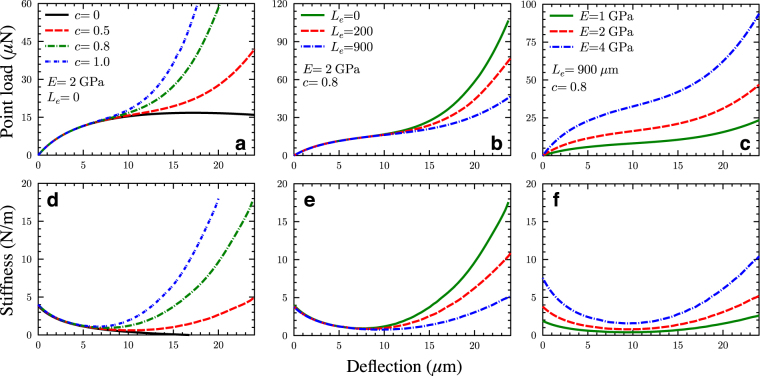
The influence of model parameters on the point-load vs. deflection curves (top row) and point-stiffness vs. deflection curves (bottom row) for the BM without a soft TBC layer (using the model version in Fig. 1c). (**a,d**) Effects of varying the volume-dispersal factor *c*. (**b,e**) Effects of varying the axial stiffness parameter *L*_*e*_ (*L*_*e*_ determines the in-plane stiffness *k*_*a*_ of the AZ and PSL). (**c,f**) Effects of varying the BM collagen-fiber elastic modulus E.

The top row of Fig. 2 shows the point load as a function of the central deflection of the BM at the basal turn (2.8 mm from the basal end) for different values of *c, L*_*e*_, and *E*. The point stiffness, which is obtained as the gradient of the load–deflection curve, is plotted in the bottom row of Fig. 2.

The case with *c* = *0* (Fig. 2a) represents the behavior of the arch-shaped lower fiber band only, in the absence of intervening ground substance, i.e., without any interaction with the upper fiber band. It is observed that the load–deflection curve varies nonlinearly for deflections of greater than twice the thicknesses of the arch (>3 μm). The slope of the point load, i.e., the stiffness, reduces with the increase in deflection and becomes zero at a critical deflection of about 17 μm (Fig. 2d), indicating buckling. Such is the behavior of an arch under point loading with end constraints^25^.

With ground substance present (c > 0), initially the load–deflection curve follows almost the same path as that of the arch alone. But as the deflection increases, the internal pressure also increases. Consequently, the upper beam layer undergoes stretching and its effective stiffness increases. For *c* ≥ 0.5, the internal pressure *p* developed in the ground substance prevents buckling instability of the arch, and the load–deflection curve increases monotonically (Fig. 2a,b,c). Thus, the point stiffness drops with deflection initially, when the arch effect is predominant, reaches a minimum, and then increases due to the quadratic stiffening of the flat beam (Fig. 2d,e,f). The minimum occurs at a much larger deflection (around 10 μm) than the physiologically relevant deflections on the order of 10 nm. The physiologically relevant point stiffness, therefore, is the linear stiffness at near-zero deflections, which is considerably higher than the stiffness at the minimum.

Figure 2d shows that the value of *c* affects the minimum of the stiffness as well as the slope of the post-minimum stiffness curve. Figure 2e shows that the effective spring length *L*_*e*_ affects the slope of the post-minimum stiffness curves, without much effect on the stiffness minimum itself, as shown in the 7–12 μm deflection range. The variations to the Young’s modulus cause differences in the point load (Fig. 2c) and stiffness (Fig. 2f) in all three deflection regions, i.e., before, during, and after the stiffness minimum.

With this as background for our analysis, we next look at the effects of a layer of TBCs on the ABM deflection mechanics.

### Effects of tympanic border cells on point-load stiffness and comparisons with experiments

**Figure 3:**
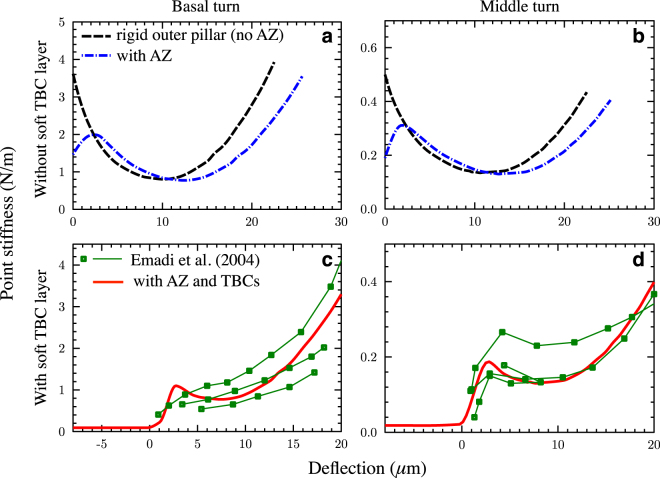
Point-stiffness vs. deflection plots from the ABM, without (**a,b**) and with (**c,d**) the soft TBC layer. The left column (**a,c**) is for the basal turn (2.8 mm from the stapes), while the right column (**b,d**) is for the middle turn (6 mm from the stapes). (**a,b**) Effects of a rigid outer pillar (black dashed lines; using the model in Fig. 1c) and of the full model (Fig. 1d) with an AZ and compliant outer-pillar support (blue dash-dotted lines). (**c,d**) The first plateau (<0 μm deflection) of the model with TBCs (red lines; using the model in Fig. 1d) is due to the compression of the soft TBC layer, which delays the buckling behavior of the arch until near the second plateau (6–10 μm deflection). The stiffness of the second plateau and of the following region of quadratic stiffening corresponds well to measurements at both locations (thin green lines with square markers from ref. 24). The key model parameters are *L*_*e*_ = 1400 μm and 3800 μm for the basal and mid-turn locations, *c* = 0.87, and *E* = 2 GPa.

Figure 3 shows the stiffness calculations for the gerbil ABM without (top row) and with (bottom row) the TBCs, at the basal turn (2.8 mm from the basal end; left column) and middle turn (6 mm from the basal end; right column). The diameter of the measurement probe *d* is taken as 25 *μ*m for the calculations, which is equal to the diameter of the probe used by Emadi *et al.* ^24^ for point-load stiffness measurements.

Without the TBCs and with a rigid outer pillar (Fig. 3a,b; black dashed lines), using the model shown in Fig. 1c, the point stiffness starts at a high value and decreases as the deflection increases, reaching a local minimum in the 10 μm region, and then increases again as the deflection increases further. The starting value of the stiffness at the basal end is 3.73 N/m, while for the middle turn it is 0.50 N/m.

When the nonlinear bending of the AZ and compliance of the outer-pillar support of stiffness *k*_*o*_, as shown in Fig. 1d, are incorporated (blue dash-dotted lines), then the initial stiffness has a lower starting value of 1.6 and 0.21 N/m for the basal and mid turns respectively. But with deflections beyond 2–3 μm, the AZ becomes stiffer against bending and the point-load stiffness becomes similar to the rigid-outer-pillar case, but with a rightward shift in the curve (Fig. 3a,b).

The point stiffness as a function of probe depth for the gerbil PZ (Fig. 3c,d) exhibits some common features between the model with TBCs (red solid lines; using the model shown in Fig. 1d) and results reported by Emadi *et al.* ^24^ (the individual thin green lines with square markers are from different preparations), namely: (i) a long initial “first plateau” stiffness region before the reference displacement of 0 μm; (ii) a sudden rise in stiffness with further displacement of the probe; (iii) a local minimum (“second plateau”), promptly followed by (iv) a quadratic increase in stiffness. The quadratic increase in stiffness is a well-known characteristic of beam behavior under a large deformation due to point or pressure loads^17^.

It is evident that the TBC layer, being much softer than the arch, deflects the most until it flattens fully, beyond which only the arch deflects. Thus, the TBC layer plays the role of obscuring the actual stiffness–deflection curve of the BM until it is fully compressed. This explains the long flat region (with a marginally negative slope), spanning over the initial deflection. This effect has been observed consistently in the measurements by Emadi *et al.* ^24, 26^ and Mountain *et al.* ^6, 22^, but they described it as noise, possibly because the behavior is contrary to the expectation of a constant stiffness followed by a monotonically increasing stiffness as predicted by the SBM.

The thickness *t*_*TBC*_ and spring stiffness *k*_*TBC*_ of the TBC layer were chosen so as to obscure the stiffness–deflection curve until near the rise in stiffness, in line with the experimental observations. This was achieved by defining the product *t*_TBC_*k_*TBC*_ to be 12 *μ*N for the basal turn and 1.9 *μ*N for the middle turn.

It can be seen from the bottom row of Fig. 3 that the predicted second-plateau stiffness on most occasions closely matches those of the measurements of Emadi *et al.* ^24^. The predicted slope in the post-second-plateau region above about 10 μm of deflection is in good agreement with the measurements, considering their variability (green curves). This match was obtained using an effective spring length *L*_*e*_ of 1400 μm and 3800 μm for the basal and mid-turn locations, respectively, and a volume dispersal factor *c* = 0.87 for both locations. The large values of *L*_*e*_ essentially indicate a small value of the SSL stiffness.

The Young’s modulus *E* of the collagen fibers was taken as 2 GPa, which is close to the value reported by Miller^7^ (1.8 GPa). The same values of *E* and c also work for the apical location (10.3 mm from the base), where the predicted mid-pectinate second-plateau stiffness is also in excellent agreement with the measurements (Table S2).

### Summary of the physiologically relevant stiffness

The stiffness without the TBCs near zero deflection in Fig. 3a,b (blue dash-dotted line) represents the point stiffness of the BM that determines the frequency–place map at the mid-pectinate zone, as actual physiological deflections of the BM are in the range of *~*10 nm only. It is, however, evident from Fig. 3c,d (<0 μm) that, for this range of deflections and beyond, the stiffness versus deflection behavior is completely obscured by the first plateau caused by compression of the TBCs.

### Pressure-Load Behavior

#### Radial deflection profile

The BM response to pressure loading was obtained using the same value for the Young’s modulus of the fiber layers as in the point-load case (2 GPa). The total BM width *L*, the sum of the PZ width *L*_*p*_ and AZ width *L*_*a*_ (*L* = *L*_*p*_ + *L*_*a*_), were taken from Schweitzer *et al.* ^5^ for the different locations along the cochlea, applying linear interpolation. The ratio *L*_*a*_ */L*_*p*_ was taken as 0.4, similar to the values of 0.39 taken by Cooper^27^ and 0.43 by Miller^7^. The other geometric parameters are the same as for the point-load case.

**Figure 4:**
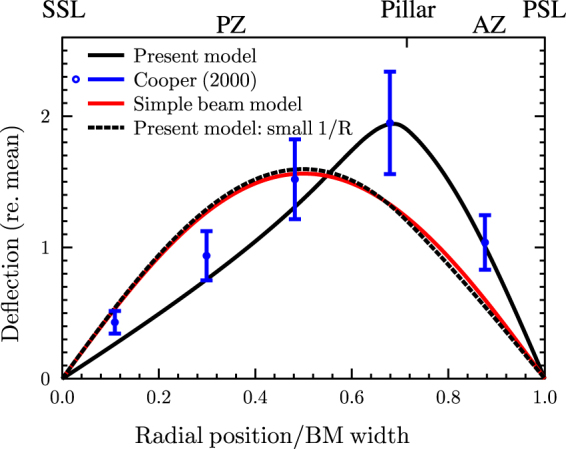
Comparison of predicted BM deflection profiles under pressure load for the ABM (using the model in Fig. 1d) as a function of normalized radial position, at the basal turn, for the present model (solid black line) and measurements from Cooper^27^ (blue circles and error bars). Also shown is the deflection profile for the SBM (solid red line) and for the ABM with a small 1/R value, corresponding approximately to a flat beam (dashed black line). The deflection is normalized by the mean deflection. The radial position is normalized by the total BM width, i.e., the distance between the PSL and SSL, as in ref. 27.

The deflection profile of the upper fiber layer across the BM width from the PSL to the SSL, as predicted by the present model (Fig. 1d with the outer-pillar spring removed), is plotted in Fig. 4 for the basal location (solid black line). From the distorted shape of the curve, it is clear that the arch substantially stiffens the PZ, such that its deflection resembles a rigid-body rotation with a hinge close to its junction with the AZ. As a result, the maximum deflection occurs at this hinge point near the connection at the outer-pillar foot. The shape of the deflection, normalized by its mean value between the PSL and SSL, agrees very well with the measurements of Cooper (blue circles and error bars) for gerbil, who observed this profile to be almost independent of the frequency of the pressure excitation^27^. Ehret and Frankenreiter^28^ observed a similar deflection profile for the house mouse.

The BM deflection profile for an SBM is also shown (Fig. 4, red line), which reaches a peak near the center of the BM and is not consistent with Cooper’s measurements. With a large radius of curvature R (small 1/R), the ABM is shown to approximately match the SBM (dashed black line), which speaks to the generalizability of the present mathematical formulation.

#### Frequency–place cochlear map

**Figure 5:**
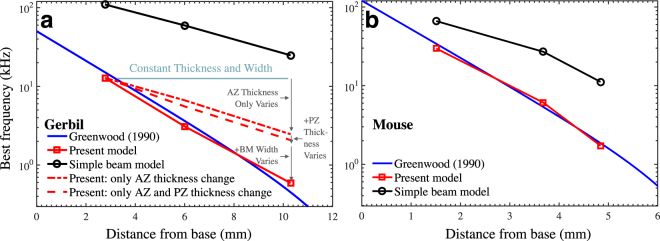
(**a**) Comparison of the predicted frequency–place cochlear map of the gerbil cochlea from the ABM (solid red line; using the model in Fig. 1d) and SBM (solid black line) under pressure loading. Also shown is the Greenwood cochlear map^29^ (blue line). The red dash-dotted line shows the effects of varying only the AZ fiber-layer thickness longitudinally with position in the ABM. The red dashed line shows the effects of varying the thickness of both the AZ and PZ fiber layers longitudinally with position. For the solid red (ABM) and black (SBM) lines, both the BM fiber-layer width and thickness vary longitudinally with position. Since the AZ width is proportional to the total width, the difference between red solid and dash-dotted lines is due primarily to the AZ width variation. (**b**) Comparison of the SBM and ABM for the mouse cochlea to the corresponding Greenwood cochlear map (blue line).

The volume compliance *C* of the BM is computed using SI Eq. (31), based on the deflection profile of the upper layer under pressure loading. The compliance (C) and best frequency (BF) (computed at the basal, middle, and apical locations) are listed in Table S3 and the BF is shown in Fig. 5a.

Greenwood presented a function relating the cochlear location to the BF, which was shown to be valid for several mammalian cochleae^29^. This function is plotted in Fig. 5a (solid blue line) and is compared with the present model predictions at three locations (solid red line with square markers).

The match between the ABM and Greenwood’s cochlear map is reasonable, keeping in mind that the present prediction is for a passive cochlea, whereas Greenwood’s relation corresponds to an *in-vivo* active cochlea, which has a ½-octave higher characteristic frequency due to the organ of Corti (OoC) structures^30^. The match is also good for the frequencies of 9.8 kHz and 1.8 kHz calculated from *in vitro* measurements by Emadi and Richter^26^ at 2.9 mm and 7.3 mm from the basal end, respectively. This establishes for the first time a frequency–place cochlear map for the gerbil BM based entirely on the reported anatomy. The SBM (solid black line with circular markers) with the same BM width, fiber volume fraction, and fiber-band thickness as the ABM is not able to match the measured cochlear map.

In the ABM, when changing only the AZ fiber-band thickness longitudinally and keeping all the other parameters constant (red dash-dotted line), it can be seen that the AZ thickness makes a significant contribution to the slope of the frequency–place map. When both the AZ and PZ fiber-band thicknesses are varied longitudinally, the slope of the frequency map changes only slightly (red dashed line). The arch height in gerbil shows a marginal increase from the basal to apical turn, which would result in an increase in BF from base to apex. This is contrary to an expected decrease in arch height to match the frequency reduction in that direction. Like the PZ fiber-band thickness, the arch height too does not cause any significant change in BF as long as it is sufficiently high to make the PZ rigid (not shown). The remaining contribution to the cochlear map essentially comes from the addition of longitudinal variation of the BM width (changing from the red dash-dotted line to the red solid line), because the AZ width was set to be proportional to the BM width (by a factor of 0.4). In summary, the cochlear map in the Mongolian gerbil is governed primarily by the morphological parameters of the AZ.

### Application of the ABM to the mouse

When the SBM is applied to the mouse model, it does not result in the expected frequency–place tonotopic mapping when realistic anatomical parameters are chosen (Table S4; Fig. 5b, black line with circular markers). Though not as pronounced, the mouse BM also exhibits an arched lower BM layer^28^. When the proposed ABM is used, the frequency–place map (solid red line with square markers) is more consistent with the frequency–place map based on measurements (blue line)^29^.

## Discussion

The present analysis has been for a gerbil BM without loading from the OoC. For pressure loading, only about half of the BM stiffness comes from the OoC^7, 22^, and most of this comes from the stiffness of the coupled pillar heads^23, 31^. Removing the OoC with a detergent shifts the peak of the traveling wave down by about 3/4 an octave^32^, and it has been shown that the BM point-load stiffness does not change postmortem^6^. In summary, while the frequency–place cochlear map can be shifted due to the OoC impedance, its gradient is set by the mechanics of the unloaded BM.

It has previously been argued^6, 24^ that the second plateau in the measured gerbil BM stiffness–deflection curves under point loading (Fig. 3c,d around 7 μm) represents the physiologically relevant stiffness. However, it is well known that the normal physiological motion of the BM of a healthy cochlea is in the submicron range (~10 nm), even for a high level of sound input^33^, whereas the second plateau occurs for deflections in the 5–10 μm range, which is not physiologically relevant.

In the present work, a new ABM is described, the analyses of which provide a new interpretation of published point-load stiffness measurements. By allowing the effects of compressing the soft TBC layer to be accounted for and turned on only for point-load stiffness measurements (dashed blue lines in Fig. 3a,b), the ABM allows estimation of the stiffness of the BM within the sub-micron physiological region, which was not possible before.

Because of the difficulty in completely removing the TBCs experimentally, it may not be possible to directly measure the stiffness of the BM using point-load measurement techniques, since they obscure the low-deflection physiological region of the stiffness–deflection curve (Fig. 3c,d). Recent measurements of pressure close to the gerbil BM surface, combined with measurements of the BM velocity, provide a viable alternative for estimating the mechanical stiffness^34^. These measurements show a higher impedance in comparison to previous point-stiffness estimates, which is consistent with our conclusion that the stiffness in the physiological region is higher than the reported second-plateau stiffness^6, 8, 24^.

**Figure 6:**
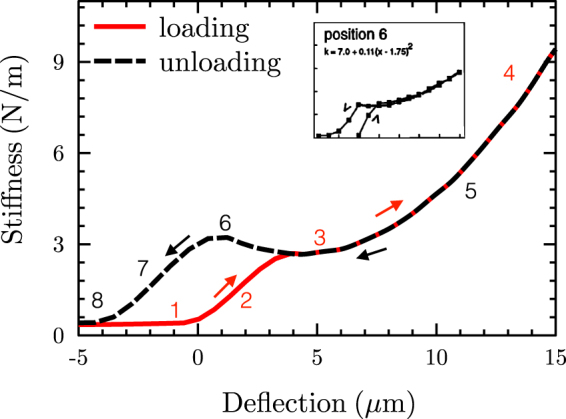
Loading (red line) and unloading (black dashed line) point-stiffness vs. deflection curves of the ABM (using the model in Fig. 1c). The ABM parameters are the same as those of Fig. 3b, but with an increase in the thickness of the arch and upper layer by a factor of 2.2 corresponding to a more basal location of 1.3 mm. The point-stiffness vs. deflection unloading curve was computed by decreasing the TBC layer thickness by 15 μm to account for the squeezing of the TBC layer by the loading cycle. The inset figure shows point-stiffness vs. deflection measurements for the loading (up-arrow) and unloading (down-arrow) conditions of the PZ at position 6, from ref. 6.

The Boston group^6, 18^ reported point-load stiffness measurements while loading and unloading the BM. For experiments with maximum displacements of less than 20 μm, they reported an unloading curve that was shifted toward lower displacements, often showing a rise toward higher stiffness values before finally dropping back down (e.g., Fig. 3 in Olson and Mountain^8^). We hypothesize that this shift during unloading is due to the soft TBC layer having been compressed as the BM was loaded by the probe, such that there can be more evidence of a buckled arch as the probe unloads the BM, since the TBC layer is already thinner. To illustrate this point, Fig. 6 shows model results for a basal location (1.3 mm from the base, corresponding to Olson’s measurement location), with the TBC layer being thicker for the loading condition than for the unloading condition.

The model results in Fig. 6 look quite similar to many of the measurements reported by the Boston group (see inset for an example). The rise in stiffness as the probe deflection decreases during unloading cannot be explained by an SBM. In summary, the initial large probe-tip movement is due to buckling of the arch, which is not seen by the upper band of the BM, and this part of the behavior is obscured by the soft tympanic cells. The upper band deflects only after this initial large displacement, and by a much smaller amount in comparison to the distance of indentation^35, 36^. This is why the overall BM structure is observed to have a much smaller displacement than the probe displacement. If not for the compression of the TBCs (Fig. 6) and the buckling of the arch (Fig. 2d), such large indentations would cause the thin fiber bands to rupture and become unable to produce the observed quadratic stiffening at higher deflections.

In the gerbil, the onset of cochlear function takes place at about 12–13 postnatal days, and during the following week it is known that there is an increase in the BF for a given BM location^5^. Müller^37^ recorded in the gerbil BM, between postnatal day 12 and postnatal day 18, a developmental shift in BF by a factor of 1.9 (0.9 octaves) at 2.9 mm from the basal end. Emadi and Richter^26^ measured the BF shift between 12 and 30 days (adult) after birth as a factor of 2 (1.0 octave) for the same location. Anatomical measurements from Schweitzer *et al.* ^5^ revealed that only the thickness of the fiber bands undergoes a significant change from infant to adult stages, while the other dimensions remain mostly the same. Interpolations of the Schweitzer data at 1.2 and 4.2 mm to approximately 2.9 mm from the basal end show that the thickness of the upper and lower bands is 0.96 μm and 0.70 μm, respectively, on 12–13 postnatal days. These fiber-band thickness values increase by a factor of about 1.5 from birth to adulthood. In the present ABM, the BF of the BM at this location with a fiber-band thickness for an infant gerbil comes out to be 7.6 kHz, whereas with the adult fiber-band thickness it comes out to be 12.8 kHz (Table S3). This corresponds to a developmental shift by a factor of 1.7, which is close to value of 1.9 measured by Müller^37^.

It is well established that for most mammalian cochleae (e.g., Table S1 for guinea pig), the BM width increases, BM thickness decreases, and BM collagen-fiber volume fraction increases gradually from base to apex, leading to the tonotopic organization of hearing consistent with an SBM^2, 16, 17, 21^. We presently hypothesize that the dominant factors that determine the cochlear map in the Mongolian gerbil, Meriones Unguiculatus (MU), are the width and thickness of the AZ beam and the collagen-fiber volume fraction, with the PZ arch having a high-enough stiffness that its flexing deformation can be considered somewhat insignificant (Fig. 4).

MU is one of the species of the subfamily Gerbillinae from the Rodentia order of mammals. Plassmann *et al.* ^13^ reported the total BM width, PZ thickness, and AZ thickness of five Gerbillinae species, including MU. They showed that in all five species, the BM width increases, but only moderately, between 15% and 90% of the distance from base to apex (Fig. S1), while the AZ thickness decreases systematically from base to apex (Fig. S3). It is notable that, in all but one of the species, the PZ thickness dramatically rises and then falls from base to apex (Fig. S2), which in the SBM formulation would result in a non-monotonic cochlear map (Eq. 1). However, as Fig. 5a shows, based on the ABM formulation of the cochlear map, the contribution of the irregularly varying PZ thickness should be minimal in comparison to that of the more regularly varying AZ thickness and BM width.

Previous estimates of the presence of an arch in non-rodent mammals might be underestimated because of dehydration of the hyaline matrix between the collagen-fiber layers resulting during specimen preparation. More work is needed using imaging methods that do not distort the BM morphology to evaluate this in greater detail.

In mammalian fossil records, the change in BM width from base to apex is estimated by the distance between the PSL and SSL (if those structures survive), because soft tissues like the collagen fibers of the BM are no longer evident in these specimens. It is possible that the estimated BM width for some of these early mammalian fossils might exhibit only moderate variation along the cochlear length, similar to that of gerbil, in which case an arch–beam BM might be the dominant mechanism for determining the frequency–place cochlear map.

## Methods

### Point-load mechanics

Figure 1c shows a simplified version of the ABM without the AZ and with no TBC layer, to highlight the arch mechanics of the PZ in isolation. The lower fiber band is modeled as a segmental arch with radius R. The lower arch thickness t_1_ and upper flat beam thickness t_2_ are from Schweitzer *et al*.^5^. Other anatomical dimensions, namely the PZ width *L*_*p*_ and the height *h*, for computing the BM stiffness under a point load at the mid-point of the arch (Q in Fig. 1c), are taken from Edge *et al*.^14^. The model parameters at three locations: basal, middle, and apical, are listed in Table S2.

The arch and beam are coupled by the intervening ground substance, which acts as an incompressible gel and is modeled as a solid with a very low elastic modulus and a Poisson’s ratio close to 0.5. As a good approximation, the ground substance is modeled as a uniform internal fluid pressure, marked as *p* in Fig. 1c, with a conserved volume (area).

With a localized point load, some of the ground substance will displace in the longitudinal direction, which reduces the cross-sectional area. The fraction of the remaining area of ground substance is denoted by the volume-dispersal factor *c*. A detailed analysis of this indicates that a value of 0.7–0.9 for *c* is reasonable (not shown).

The boundary conditions of the upper fiber band are such that the ends stretch in the horizontal (*X*) direction with an effective stiffness of *k*_*a*_, parameterized in terms of a length dimension *L*_*e*_ (*k*_*α*_ = *Eb* (*t*_1_ + *t*_2_)/*L*_*e*_, where *E* is the elastic modulus, *b* is the effective longitudinal breadth of the model, and *t*_*1*_ and *t*_*2*_ are the respective thicknesses of the lower and upper fiber bands), which represents the in-plane stiffness of the AZ and SSL.

The point-load stiffness measurements show that the highest point stiffness of the BM occurs at the outer pillar^6, 7, 22^, which implies a significant restraint to the BM deflection. This restraint was modeled using a spring support by Miller^7^. On the other hand, Cooper^27^ observed that it is this very location beneath the foot of the outer pillar that experiences the near maximal deflection under pressure loading. This would not be possible with the presence of a stiff support at this location. Steele *et al.* ^31^ noted this difference in the behavior of the BM under point and pressure loading, and were able to match their computations with the measured BM radial displacement profiles by including a spring support due to the outer pillar in the case of point loading (as in Fig. 1d) and removing it for the case of pressure loading. Both Miller^7^ and Steele *et al.* ^31^ argued that the heads of the adjacent pillars are connected to each other along the length of BM with a rather high coupling stiffness. This restrains the pillars from rotation at one location of the BM relative to another, as is required under point loading, and hence increases the stiffness. The restraint becomes negligible under pressure loading when the neighboring pillars move together. Accordingly, the outer-pillar spring shown in Fig. 1d is removed for pressure loading.

The outline of the mathematical formulation of the ABM is described below, with the details provided in the Supplemental Information (SI).

### The simple beam model (SBM)

In an SBM, the local resonance frequency *f*_*local*_ at a point along the length of the BM is affected by the local fiber volume fraction *v*_*f*_ (assuming a uniform distribution of collagen fibers), the local BM thickness *t* (assuming uniform thickness across the BM width), the fluid density *ρ*_*f*_, and the local BM PZ width *L*_*p*_, according to the following proportionality relationship:

1 $${f}_{local}\propto {({v}_{f}{t}^{3}/{\rho }_{f}{L}_{p}^{5})}^{1/2}.$$

### Description of the arch-beam model (ABM)

The geometry of the general ABM of the BM is shown in Fig. 1d. The curved lower band in the PZ is modeled as a shallow segmental arch of thickness *t*_*1*_, height *h*, and span *L*_*p*_, and the upper band as a beam of thickness *t*_*2*_. The arch and beam are coupled together through the soft ground substance, which acts as an incompressible gel. In the AZ, the two layers merge to form a beam of combined thickness *t*_*1*_ + *t*_*2*_. The transverse deflection *w* is restrained at the bony PSL and SSL endpoints, since these are much stiffer than the BM in the transverse direction. However, the elasticity of the PSL and SSL may allow them to move in-plane in the radial direction *X*. This is modeled by restraining the in-plane displacement at the SSL end and adding an axial spring of constant *k*_*s*_ at the PSL end.

### Models for point-load measurements

An analytical formulation has been developed to predict the large-deflection response of the BM under point-load measurements. First the support due to the outer pillar was considered to be rigid (Fig. 3a,b, black lines, using the model in Fig. 1c). Figure 1c shows this simplified model, in which *k*_*a*_ represents the effective spring stiffness of the AZ and the PSL. Subsequently (Fig. 1d), the reaction on the outer pillar was applied to the upper flat beam, the AZ was included, and an additional spring support *k*_*o*_ at the outer pillar was added to model the restraint from the pillar heads (Fig. 3a,b, blue lines, using the model in Fig. 1d). The ground substance within the arch is modeled as an incompressible gel, which has the effect of providing a uniform internal pressure *p* on the arch (lower fiber band) as well as on the flat beam (upper fiber band). The effective longitudinal breadth *b* of the model is taken as equal to the diameter of the probe, since there is negligible longitudinal coupling between adjacent collagen fibers^7^.

The SI section describes the development of the equations for the ABM. A moderate-rotation geometric nonlinearity due to large deflections describes the shallow circular arch (SI Eqs. 1–22). The flat beam representing the upper band is subjected to a uniform pressure and axial tension with governing equations of equilibrium (SI Eqs. 23–30). The deformations of the arch and the beam areas are coupled by the fact that their cross-sectional area displacements, A_s_ and A_b_ respectively, are related to each other. However, under point loading, some amount of the ground substance in the loaded region may be pushed away to longitudinally adjacent areas. To account for this, the area displacements are related as A_b_ = cA_s_, with c ≤ 1 (SI Eq. 29). Finally, the mechanics of the soft TBC layer (Fig. 1b) are described by SI Eq. 30.

### Model for pressure-load behavior

A linear analysis of the pressure-load behavior was performed on the model shown in Fig. 1d with the outer-pillar support removed, using COMSOL Multiphysics software. For the lower and upper fiber layers, curved and flat beam elements were used, and for the ground substance plane-stress elements were used. The ground substance was modeled as a solid with low elastic modulus and a Poisson’s ratio of close to 0.5. The volume compliance due to a differential pressure across the BM was calculated, and from this the resonance frequencies for the reported cochlear maps (Fig. 4) were determined. The SI text and Equations 31 and 32 provide further details.

## Electronic supplementary material

Supporting Information
