# Supplementary material for: Unraveling the mystery of hearing in gerbil and other rodents with an arch-beam model of the basilar membrane
Source: Sci Rep. 2017 Mar 22;7:228. doi: 10.1038/s41598-017-00114-x (PMC5427805; doi:10.1038/s41598-017-00114-x)
Supplement: Supplementary file 1 — Supporting Information [file 41598_2017_114_MOESM1_ESM.pdf]

Unraveling the mystery of hearing in gerbil and other rodents with an arch-beam model of the basilar membrane

Santosh Kapuria[a], Charles R. Steele[b] and Sunil Puria[b,1]

[a]Department of Applied Mechanics, Indian Institute of Technology Delhi, Hauz Khas, New Delhi 110016, India

[b]Department of Mechanical Engineering, Stanford University, CA 94305, USA

Supporting Information

Methods, Tables and Figures

1. The model for point-load measurements

An analytical formulation is developed to predict the large-deflection response of the BM under point-load measurements. For mathematical simplicity, the support due to the outer pillar is considered to be stiff enough to allow any deflection, without losing much accuracy for the deflection at the center of the arch. Figure 1C shows the resulting simplified model, where k_a represents the effective spring stiffness of the arcuate zone and the SSL. The ground substance is modeled as an incompressible gel with the effect of providing a uniform internal pressure p on the arch (lower fiber band) as well as the flat beam (upper fiber band). The effective diameter d of the model is taken as equal to the diameter of the probe, since there is negligible longitudinal coupling between adjacent filaments[1].

1.1 Lower fiber band: the circular arch

It is appropriate to consider von Karman-type geometric nonlinearity due to large deflections for the present purpose. For the shallow circular arch, with circumferential axis θ and thickness axis z, the Langrangian strain ε_θ at a point $P(\theta, z)$ is given by[2]

$$\varepsilon_\theta = \frac{u_{0,\theta}}{R} + \frac{w_0}{R} + \frac{1}{2}\left(\frac{w_{0,\theta}}{R}\right)^2 - z\frac{w_{0,\theta\theta}}{R^2} = \varepsilon_0 + z\kappa, \tag{1}$$

where a subscripted comma denotes a partial differentiation. u_0 and w_0 are the in-plane and transverse displacements of the middle surface ($z = 0$) along θ and z directions, respectively. ε_0 and κ denote the membrane strain at the arch's midsurface and its curvature, respectively. R is the radius of the midsurface of the arch. Neglecting the transverse shear strain ($\gamma_{\theta z} \cong 0$) as per Love's thin shell theory assumptions, the principle of virtual work for the arch with a point load Q applied at its center, reduces to

$$\int_V \sigma_\theta \delta\varepsilon_\theta dV + \int_S bp\delta w_0 - Q\delta w_c = 0 \tag{2}$$

for all kinematically admissible virtual displacements δu_0 and δw_0. V and S denote, respectively, the volume and arc length of the arch. σ_θ denotes the circumferential normal stress and w_c is the central deflection at $\theta = 0$. Substituting Eq. (1) into Eq. (2) and performing integration over the thickness of the arch yields

$$\int_S \left[N_\theta\left(\delta u_{0,s} + \frac{1}{R}\delta w_0 + w_{0,s}\delta w_{0,s}\right) - M_\theta \delta w_{0,ss} + \bar{p}\delta w_0\right] ds - Q\delta w_c = 0, \tag{3}$$

where $s = R\theta$, $\bar{p} = bp$, and N_θ and M_θ are the membrane and bending stress resultants, respectively, given by

$$N_\theta = \int_{-t_1/2}^{t_1/2} b\sigma_\theta dz \quad \text{and} \quad M_\theta = \int_{-t_1/2}^{t_1/2} bz\sigma_\theta dz. \tag{4}$$

---

[1] Corresponding author: Sunil_Puria@meei.harvard.edu

The integral in Eq. (3) is expressed in terms of variations δu_0 and δw_0 by applying integration by parts when necessary, which yields

$$\int_S \left[-N_{\theta,s}\delta u_0 + \frac{N_\theta}{R}\delta w_0 - \left(N_\theta w_{0,s}\right)_{,s}\delta w_0 - M_{\theta,ss}\delta w_0 + \bar{p}\delta w_0\right] ds$$

$$+\left[N_\theta \delta u_0 + \left(N_\theta w_{0,s}\delta w_0 + M_{\theta,s}\right)\delta w_\theta - M_\theta \delta w_{0,s}\right]_S - Q\delta w_c = 0. \qquad (5)$$

Since the virtual displacements are arbitrary, their coefficients in the integrand of Eq. (5) must vanish separately. This yields the governing equations of equilibrium of the arch under large deflection:

$$N_{\theta,s} = 0, \quad \Rightarrow \qquad N_\theta = \text{constant} = \pm \bar{N}_1,\ \bar{N}_1 > 0; \qquad (6)$$

$$M_{\theta,ss} - \frac{N_\theta}{R} + \left(N_\theta w_{0,s}\right)_{,s} + \bar{p} = 0. \qquad (7)$$

The variationally consistent boundary conditions are the prescribed values of one of the factors of each of the following products appearing in Eq. (5):

$$N_\theta \delta u_0, \qquad \left(N_\theta w_{0,s} + M_{\theta,s}\right)\delta w_0, \ \text{and} \quad M_\theta \delta w_{0,s}. \qquad (8)$$

In the present problem, considering symmetry, the boundary conditions are

$$\text{at } s = 0: \qquad w_{0,s} = 0, \qquad M_{\theta,s} = Q/2, \qquad \text{and}$$

$$\text{at } s = \pm S/2: \qquad w_0 = 0, \qquad M_\theta = 0. \qquad (9)$$

From Eq. (6), N_θ is constant, which can be compressive or tensile depending on the relative values of Q and p.

Case 1: $N_\theta < 0$

From its definition in Eq. (4), M_θ can be related to w_0 as $M_\theta = -EI_1 w_{0,ss}$, where I_1 is the moment of inertia of the arch cross-section about its neutral axis along the direction perpendicular to the *θ*–*z* plane. Substituting this relation into Eq. (7) yields

$$w_{0,ssss} + \mu_1^2 w_{0,ss} = \mu_1^2 \bar{p}_1/R, \qquad (10)$$

where $\mu_1^2 = \bar{N}_1/EI$ and $\bar{p}_1 = (\bar{p}R + \bar{N}_1)/\bar{N}_1$. Its solution satisfying the boundary conditions can be obtained as

$$w_0 = -\frac{\bar{Q}}{\mu_1^2 R \bar{s}_0}\{\tan\bar{s}_0 \cos\bar{s} - \bar{s}_0 - H(s)(\sin\bar{s} - \bar{s})\} - \frac{\bar{p}_1}{\mu_1^2 R}\left\{1 - \frac{\cos\bar{s}}{\cos\bar{s}_0} + \frac{\bar{s}_0^2}{2} - \frac{\bar{s}^2}{2}\right\}, \quad (11)$$

where $\bar{s} = \mu_1 s$, $\bar{s}_0 = \mu_1 S/2$, $\bar{Q} = QRS/4EI$, and $H(s)$ is the Heaviside step function defined by $H(s) = \begin{Bmatrix} -1 \text{ when } s < 0 \\ 1 \text{ when } s \geq 0 \end{Bmatrix}$. The central deflection w_c at $s = 0$ is obtained from Eq. (11) as

$$w_c = -\frac{\bar{Q}}{\mu_1^2 R \bar{s}_0}\{\tan\bar{s}_0 - \bar{s}_0\} - \frac{\bar{p}_1}{\mu_1^2 R}\left\{1 - \frac{1}{\cos\bar{s}_0} + \frac{\bar{s}_0^2}{2}\right\}. \qquad (12)$$

The area displacement (A_s) of the arch is obtained as

$$A_s = \int_{-S/2}^{S/2} w_0 ds = -\frac{2\bar{Q}}{\mu_1^3 R \bar{s}_0}\left\{\frac{1}{\cos\bar{s}_0} - \frac{\bar{s}_0^2}{2} - 1\right\} - \frac{2\bar{p}_1}{\mu_1^3 R}\left\{\bar{s}_0 - \tan\bar{s}_0 + \frac{\bar{s}_0^3}{3}\right\}. \quad (13)$$

Since N_θ is constant, the membrane strain ε_0 can be computed as $\varepsilon_0 = -\bar{N}_1/Ebt_1 = -\mu_1^2 r_1^2$, with $r_1 = \sqrt{I_1/bt_1}$. Integrating this and noting that $u_0(-S/2) = 0$ yields the lateral displacement at the outer-pillar end ($s = S/2$) as

$$u_0(S/2) = -\mu_1^2 r_1^2 S - \frac{1}{R}\int_{-S/2}^{S/2} w_0 ds - \frac{1}{2}\int_{-S/2}^{S/2} (w_{0,s})^2 ds, \qquad (14)$$

which should be equal to the deformation of the spring of stiffness k_a:

$$u_0(S/2) = -k_a(N_1 - N_2), \qquad (15)$$

where N_2 is the tensile force in the flat beam. We introduce an effective length L_e for the spring constant k_a such that $k_a = Eb(t_1 + t_2)/L_e$. Substituting the expression of w_0 from Eq. (11) into Eqs. (14) and (15) yields

$$A_1\bar{Q}^2 + B_1\bar{Q} + C_1 = 0, \tag{16}$$

where

$$A_1 = \frac{1}{4\bar{s}_0^4}\left[3 - \frac{3\tan\bar{s}_0}{\bar{s}_0} + \tan^2\bar{s}_0\right],$$

$$B_1 = \frac{1}{\bar{s}_0^4}\left[\frac{2\bar{p}_1 - 1}{\cos\bar{s}_0} - (2\bar{p}_1 - 1) + \left(\frac{1-\bar{p}_1}{2}\right)\bar{s}_0^2 - \frac{\bar{p}_1\bar{s}_0\tan\bar{s}_0}{2\cos\bar{s}_0}\right], \tag{17}$$

$$C_1 = \left(\frac{\bar{s}_0}{\lambda_s}\right)^2\left(1 + \frac{t_1}{t}\frac{L_e}{S}\right) + D_1 - 4\frac{N_2}{Ebt}\frac{L_eR^2}{S^3}, \text{ and}$$

$$D_1 = \frac{1}{4\bar{s}_0^2}\left[(5\bar{p}_1^2 - 4\bar{p}_1) + \frac{2}{3}(\bar{p}_1^2 - 2\bar{p}_1)\bar{s}_0^2 + (4\bar{p}_1 - 5\bar{p}_1^2)\frac{\tan\bar{s}_0}{\bar{s}_0} + \bar{p}_1\tan^2\bar{s}_0\right],$$

with $\lambda_s = S^2/4r_1R$ and $t = t_1 + t_2$.

Case 2: $N_\theta > 0$

In this case, $N_\theta = \bar{N}_1$, with $\bar{N}_1 > 0$, for which the governing differential equation for w_0 becomes

$$w_{0,ssss} - \mu_1^2 w_{0,ss} = -\mu_1^2\bar{p}_1/R, \tag{18}$$

where $\bar{p}_1 = (-\bar{p}R + \bar{N}_1)/\bar{N}_1$. Equations (11)–(13) and the coefficients of Eq. (16) take the following forms:

$$w_0 = \frac{\bar{Q}}{\mu_1^2 R\bar{s}_0}\{\tanh\bar{s}_0\cosh\bar{s} - \bar{s}_0 - H(s)(\sinh\bar{s} - \bar{s})\} + \frac{\bar{p}_1}{\mu_1^2 R}\left\{1 - \frac{\cosh\bar{s}}{\cosh\bar{s}_0} - \frac{\bar{s}_0^2}{2} + \frac{\bar{s}^2}{2}\right\}, \tag{19}$$

$$w_c = \frac{\bar{Q}}{\mu_1^2 R\bar{s}_0}\{\tanh\bar{s}_0 - \bar{s}_0\} + \frac{\bar{p}_1}{\mu_1^2 R}\left\{1 - \frac{1}{\cosh\bar{s}_0} - \frac{\bar{s}_0^2}{2}\right\}, \tag{20}$$

$$A_s = \frac{2\bar{Q}}{\mu_1^3 R\bar{s}_0}\left\{\frac{1}{\cosh\bar{s}_0} + \frac{\bar{s}_0^2}{2} - 1\right\} + \frac{2\bar{p}_1}{\mu_1^3 R}\left\{\tanh\bar{s}_0 - \bar{s}_0 + \frac{\bar{s}_0^3}{3}\right\}, \tag{21}$$

$$A_1 = \frac{1}{4\bar{s}_0^4}\left[3 - \frac{3\tanh\bar{s}_0}{\bar{s}_0} - \tanh^2\bar{s}_0\right], \tag{22a}$$

$$B_1 = \frac{1}{\bar{s}_0^4}\left[\frac{2\bar{p}_1 - 1}{\cosh\bar{s}_0} - (2\bar{p}_1 - 1) - \left(\frac{1-\bar{p}_1}{2}\right)\bar{s}_0^2 + \frac{\bar{p}_1\bar{s}_0\tanh\bar{s}_0}{2\cosh\bar{s}_0}\right], \tag{22b}$$

$$C_1 = \left(\frac{\bar{s}_0}{\lambda_s}\right)^2\left(1 - \frac{t_1}{t}\frac{L_e}{S}\right) + D_1 - 4\frac{N_2}{Ebt}\frac{L_eR^2}{S^3}, \text{ and} \tag{22c}$$

$$D_1 = \frac{1}{4\bar{s}_0^2}\left[-(5\bar{p}_1^2 - 4\bar{p}_1) + \frac{2}{3}(\bar{p}_1^2 - 2\bar{p}_1)\bar{s}_0^2 - (4\bar{p}_1 - 5\bar{p}_1^2)\frac{\tanh\bar{s}_0}{\bar{s}_0} + \bar{p}_1\tanh^2\bar{s}_0\right], \tag{22d}$$

1.2 Upper fiber band: the flat beam

The flat beam representing the upper fiber band is subjected to a uniform pressure p and an axial tension N_2. Its governing equation of equilibrium can be obtained in terms of the deflection w_0^b, following the same procedure as for the arch:

$$w_{0,xxxx}^b - \mu_2^2 w_{0,xx}^b = -\mu_2^2\bar{p}_2, \tag{23}$$

where $\mu_2^2 = N_2/EI_2$ and $\bar{p}_2 = p/N_2 = p/\mu_2^2 EI_2$, with I_2 being the moment of inertia of the beam section. The solution of Eq. (23) satisfying the boundary conditions, $w_0^b = w_{0,xx}^b = 0$ at $x = L_p/2$ and $w_{0,x}^b = w_{0,xxx}^b = 0$ at $x = 0$, is obtained as

$$w_0^b = \frac{\bar{p}_2}{\mu_2^2}\left[1 - \frac{\cosh\bar{x}}{\cosh\bar{x}_0} + \frac{\bar{x}^2}{2} - \frac{\bar{x}_0^2}{2}\right], \tag{24}$$

where $\bar{x} = \mu_2 x$ and $\bar{x}_0 = \mu_2 L_p/2$. The area displacement A_b is obtained as

$$A_b = \frac{\bar{p}_2}{\mu_2^3}\left[\bar{x}_0 - \tanh\bar{x}_0 - \frac{\bar{x}_0^3}{3}\right], \tag{25}$$

Also, equating the axial displacement at $x = L_p/2$ to the shortening of the end spring k_a yields

$$\mu_2^2 r_2^2 L_p - \frac{1}{2}\int_{-L_p/2}^{L_p/2}\left(w_{0,x}^b\right)^2 dx = -\frac{(N_2 - N_1)L_e}{Ebt}, \tag{26}$$

with $r_2 = \sqrt{I_2/bt_2}$. Substituting w_0^b from Eq. (24) into Eq. (26) leads to

$$\bar{p}_2 = \frac{2\bar{x}_0^3}{\sqrt{\Gamma}}\left[\frac{\bar{x}_0^2}{\lambda_b^2} + (\mu_2^2 I_2 - \mu_1^2 I_1)\frac{L_e}{bt}\right]^{\frac{1}{2}}, \tag{27}$$

where $\lambda_b = L_p/2r_2$ and

$$\Gamma = 5\frac{\tanh\bar{x}_0}{\bar{x}_0} - \frac{1}{\cosh^2\bar{x}_0} + \frac{2}{3}\bar{x}_0^2 - 4. \tag{28}$$

Equations 1–28 are implemented in a custom MATLAB script.

1.3 Coupling of the arch and the beam

The deformations of the arch and the beam are coupled by the fact that the area displacements, A_s and A_b respectively, are related to each other. Considering incompressibility of the ground substance and total-mass conservation at a given cross section of the PZ arch, the two area displacements should be equal. However, under point loading, some amount of the ground substance in the loaded region may be pushed away to adjacent areas. To account for this, the area displacements are related as

$$\mathrm{A}_b = c\mathrm{A}_s, \qquad 0 \le c \le 1, \tag{29}$$

where c is the volume-dispersal factor. A model for this effect has been developed in which the pectinate zone is treated as an elastic tube. The result for reasonable tissue properties that c is around 0.8. However, in the present calculations, the effect of various values for c are also considered. The unknowns in the arch-beam-ground substance system are μ_1, μ_2, and $\bar{p}$, which are obtained by solving the system of nonlinear equations (16), (27), and (29). The solution is obtained iteratively using a displacement-controlled approach (finding Q for a given w_c).

1.4 Effects of the soft cell cover

The fact that the soft cells on the scala-tympani side of the BM affect the force–deflection curve under point loading has been demonstrated by Miller[1]. Like the ground substance, these cells too can be approximated as an incompressible fluid layer with a low shear strength under pressure loading. Under a point force, however, these cells would act as a soft spring, which would deflect the most until fully compressed. The final load–deflection behavior would be that of this spring and the nonlinear spring representing the load–deflection curve of the arch-beam-ground substance system, acting in series. Denoting the thickness and the spring stiffness of the soft cell layer as t_{cell} and k_{cell}, respectively, the effective deflection w_e for a given force Q will be

$$w_e = \begin{Bmatrix} Q/k_{cell} + w_c & \text{when } Q/k_{cell} < t_{cell} \\ t_{cell} + w_c & \text{when } Q/k_{cell} \ge t_{cell} \end{Bmatrix}. \tag{30}$$

2. The model for pressure-load behavior

For pressure loading, the model shown in Fig. 1D is considered without the spring support (k_o) at the outer-pillar foot. The ground substance is modeled as a solid of very low elastic modulus and a Poisson's ratio close to 0.5 (not exactly 0.5 to avoid numerical difficulties). Since the soft cell cover will act as an incompressible layer under pressure loading, it will not affect the deflection. Only the linear stiffness corresponding to the physiological range of deflections is of importance here. Hence, a linear analysis is

performed using the commercial finite-element software COMSOL. The lower and upper fiber layers are modeled using curved and flat beam elements, and the ground substance using plane stress elements.

The volume compliance C is computed as

$$C = \frac{\Delta A}{p_0}, \tag{31}$$

where ΔA is the area displacement of the BM subjected to a differential pressure p_0, computed from the deflection w as $\Delta A = \int_0^{(L_p+L_a)} w dx$. The BM is covered with soft cells that are in contact with extracellular fluid, both of which have similar density and acoustical properties. Consequently, for modeling, the soft cells can be replaced by fluid. Based on these assumptions, Puria and Steele[17] obtained the best frequency f of the BM as a plate immersed in fluid. Following this formulation, the best frequency of the BM can be estimated from its volume compliance as

$$f = \frac{1}{\pi}\sqrt{\frac{1}{\pi \rho_F C}}, \tag{32}$$

where ρ_F is the fluid density and f is in Hz.

3. SI Tables

Table S1: Predicted frequency ratios calculated from the Simple Beam Model for the guinea pig and gerbil from basal to apical ends.

	Guinea pig[3]			Gerbil		
	Basal end	Apical end	Ratio (Base/Apex)	Basal end	Apical end	Ratio (Base/Apex)
Fiber vol. fraction (v_f)[e]	0.08	0.01	8	0.16[a]	0.0083[a]	19.3
BM PZ width[f] (L_p), µm	80	180	0.44	148[b]–93.6[c]	211[b]–214[c]	0.07–0.44
BM thickness[4] (t), µm	7	1	7	22.6[b]	56.5[b]	0.40
Point stiffness[d] ratio	-	-	32213	-	-	3.6–14.5
Resonant frequency ratio	-	-	400	-	-	2.7–8.7
Auditory Range, Hz	50,000	54	926	60,000	100	600

[a]approximated from the ratio of fiber band thickness[5] to overall thickness[6]
[b]extrapolated to basal and apical ends from measurements at three locations given in Edge *et al.*[6]
[c]extrapolated to basal and apical ends from measurements at three locations given in Schweitzer et al.[5]
[d]Point stiffness $\propto v_f t^3 / \rho_f L_p^3$
[e]Taken from the fiber density measured in cat by Cabezudo[7].
[f]As an approximation, 2/3 of the total BM width is given[7]. Extrapolation of Tuedt and Richter[8] gives higher value of 100 µm at the basal end of the guinea pig.

Table S2: Geometry parameters and measured and ABM point stiffness for gerbil BM.

Location	Basal turn	Middle turn	Apical turn
Distance from base, mm	2.8	6.0	10.3
Width of PZ (L_p)[a], µm	168.0	192.0	207.3
Height (h)[a], µm	35.0	49.7	55.3
Lower fiber band thickness (t_1)[b], µm	1.49	0.90	0.45
Upper fiber band thickness (t_2)[b], µm	1.01	0.35	0.16
Measured plateau stiffness[c], N/m	0.79	0.15	0.017
ABM-Estimated plateau stiffness, N/m	0.78	0.15	0.019

[a]from Edge *et al.*[6]
[b]interpolated at given locations from measurements in Schweitzer et al.[5]
[c]$k = 0.95 \times 10^{(10.24-4.43x)/20}$, where x is the distance from the base in mm [Fig. 3 of Emadi *et al.*[9]]

Table S3: ABM volume compliance and best frequency of gerbil BM

Location	Basal turn	Middle turn	Apical turn
Distance from base, mm	2.8	6.0	10.3
Total width (L)[a], μm	175	224	287
Volume compliance, m^2/TPa	0.20	3.34	93.27
Estimated best frequency, kHz	12.8	3.11	0.59
Best frequency[b], kHz	15.1	3.64	0.45

[a]from Schweitzer et al.[5]
[b]from Greenwood[10]

Table S4: ABM volume compliance and best frequency of house mouse

Location	Basal turn	Middle turn	Apical turn
Distance from base, mm	4.3	2.13	0.97
Width of PZ (L_p)[a], μm	95	110	115
Width of AZ (L_a)[a], μm	39	51	58.5
Height (h)[a], μm	14.1	14.0	9.8
Lower fiber layer thickness, μm	1.7	0.9	0.46
Upper fiber layer thickness, μm	1.3	0.35	0.18
Volume compliance, m^2/TPa	0.037	0.86	10.8
Estimated best frequency, kHz	29.7	6.1	1.73
Best frequency[b], kHz	34.1	5.3	1.8

[a]from Keiler and Richter[11]
[b]from Greenwood[10]

4. SI Figures

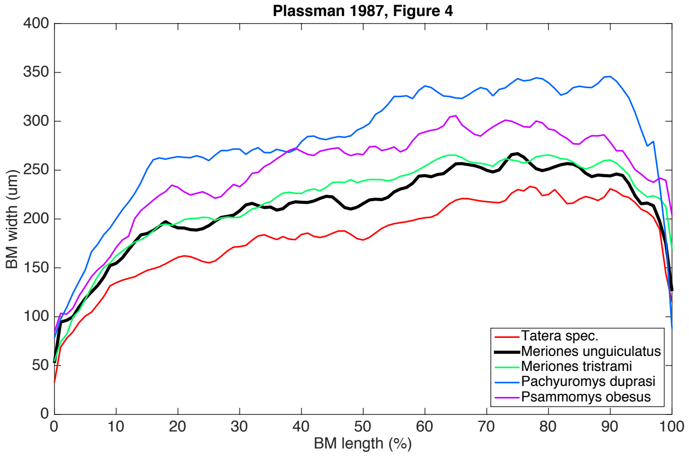

SI Figure S1: Basilar membrane total width as function of %distance from the base as measured by Plassmann *et al.*[12] for 5 species of the gerbillinae rodent family.

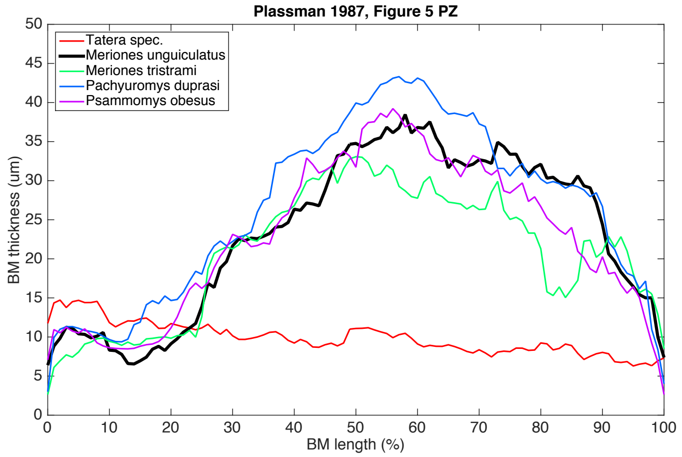

SI Figure S2: Same as SI Fig S1 but with the pectinate zone (PZ) thickness plotted.

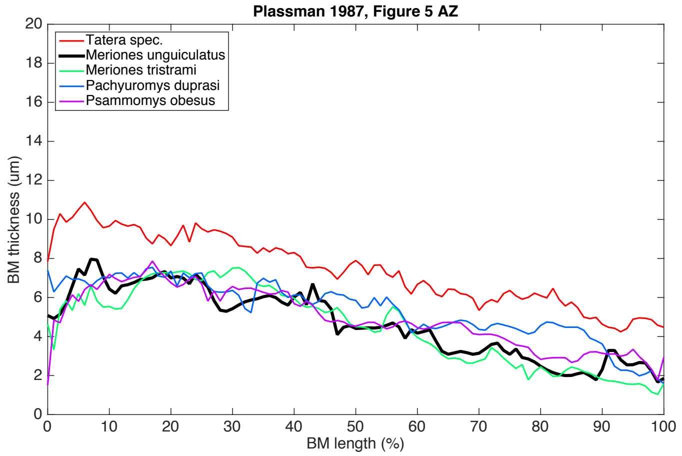

SI Figure S3: Same as SI Fig S1 but with the arcuate zone (AZ) thickness plotted.

Supporting Information References

1. Miller CE. Structural implications of basilar membrane compliance measurements. *J Acoust Soc Am* **77**, 1465-1474 (1985).
2. Bradford M, Uy B, Pi Y-L. In-plane elastic stability of arches under a central concentrated load. *Journal of engineering mechanics* **128**, 710-719 (2002).
3. Puria S, Steele CR. Mechano-acoustical transformations. (ed^(eds Dallos P, Oertel D). Academic Press (2008).
4. Fernandez C. Dimensions of the cochlea (Guinea Pig). *J Acoust Soc Am* **24**, 519-523 (1952).
5. Schweitzer L, Lutz C, Hobbs M, Weaver SP. Anatomical correlates of the passive properties underlying the developmental shift in the frequency map of the mammalian cochlea. *Hear Res* **97**, 84-94 (1996).
6. Edge RM, Evans BN, Pearce M, Richter CP, Hu X, Dallos P. Morphology of the unfixed cochlea. *Hear Res* **124**, 1-16 (1998).
7. Cabezudo LM. The ultrastructure of the basilar membrane in the cat. *Otolaryngology* **86**, ORL433 (1978).
8. Tuedt IU, Richter CP. Basilar Membrane and Tectorial Membrane Stiffness in the CBA/CaJ Mouse. *J Assoc Res Otolaryngol* **15**, 675-694 (2014).
9. Emadi G, Richter CP, Dallos P. Stiffness of the gerbil basilar membrane: radial and longitudinal variations. *J Neurophysiol* **91**, 474-488 (2004).
10. Greenwood DD. A cochlear frequency-position function for several species--29 years later. *J Acoust Soc Am* **87**, 2592-2605 (1990).
11. Keiler S, Richter CP. Cochlear dimensions obtained in hemicochleae of four different strains of mice: CBA/CaJ, 129/CD1, 129/SvEv and C57BL/6J. *Hearing research* **162**, 91-104 (2001).
12. Plassmann W, Peetz W, Schmidt M. The cochlea in gerbilline rodents. *Brain Behav Evol* **30**, 82-101 (1987).
